# Laparoscopically assisted aneurysmorrhaphy for iliac artery aneurysms following endovascular repair: reports of two cases

**DOI:** 10.1093/jscr/rjae684

**Published:** 2024-11-05

**Authors:** Kotaro Mukasa, Yasunori Yakita, Musashi Tsuda, Shinichiro Abe, Soichi Asano

**Affiliations:** Department of Cardiovascular Surgery, Chiba Cardiovascular Center, 575 Tsurumai, Ichihara, Chiba 290-0512, Japan; Department of Cardiovascular Surgery, Chiba Cardiovascular Center, 575 Tsurumai, Ichihara, Chiba 290-0512, Japan; Department of Cardiovascular Surgery, Chiba Cardiovascular Center, 575 Tsurumai, Ichihara, Chiba 290-0512, Japan; Department of Cardiovascular Surgery, Chiba Cardiovascular Center, 575 Tsurumai, Ichihara, Chiba 290-0512, Japan; Department of Cardiovascular Surgery, Chiba Cardiovascular Center, 575 Tsurumai, Ichihara, Chiba 290-0512, Japan

**Keywords:** iliac artery aneurysm, aneurysmorrhaphy, laparoscopy

## Abstract

Endovascular aneurysm repair (EVAR) is commonly utilized for iliac artery aneurysms (IAA), yet some cases necessitate additional intervention due to aneurysm re-expansion. We report two cases: a 68-year-old male with a left internal IAA (IIAA) and an 80-year-old female with both a left common iliac artery aneurysm (CIAA) and IIAA, who underwent aneurysmorrhaphy following initial EVAR. Both procedures were successful, significantly reducing aneurysm size without immediate complications. The use of laparoscopy in aneurysmorrhaphy enhances oversight of inflow vessels and facilitates suturing in deep pelvic areas. Our cases indicate that incorporating laparoscopy can substantially improve surgical outcomes.

## Introduction

Aneurysmorrhaphy following endovascular aneurysm repair (EVAR) for abdominal aortic aneurysms (AAA) is a viable option for managing AAA re-expansion [1]. However, reports on aneurysmorrhaphy after endovascular treatment for IAA are rare. Exposing the iliac artery in the deep pelvis is challenging due to visual and spatial limitations. Additionally, the proximity of the rectum, other intestinal structures, and the ureter complicate the open surgical procedure. We performed two cases of open aneurysmorrhaphy to safely control the expansion of iliac artery aneurysms (IAA) after endovascular treatment, utilizing laparoscopy. Using laparoscopy proved beneficial for observing the aneurysm’s interior, enhancing the overall quality of the surgical procedure.

## Case 1

**Figure 1:**
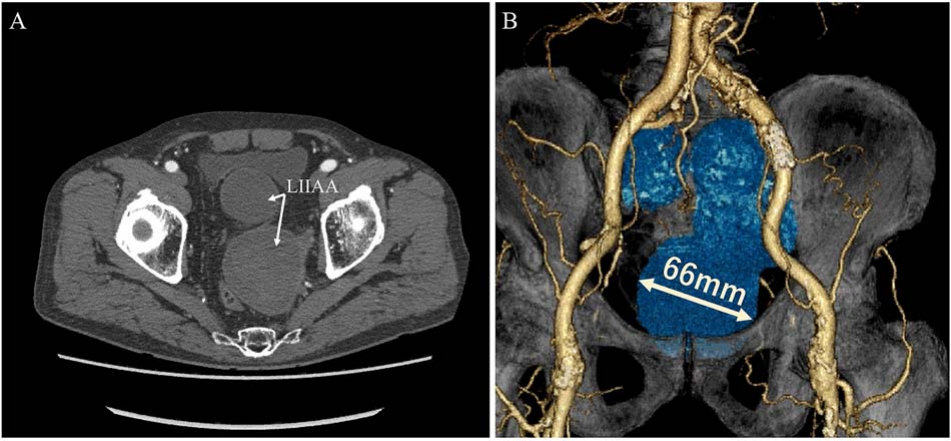
Preoperative computed tomography imaging showing a giant left internal iliac artery aneurysm in a dumpling-like cluster with a maximum short diameter of 66 mm. A—Axial image, B—3D image. LIIAA, left internal iliac artery aneurysm.

A 68-year-old male was admitted for the expansion of a left IIAA. He had undergone coiling of peripheral branches and stent graft placement in the left common iliac artery (CIA) 10 years prior. Eight years prior, an endoleak was detected between the left CIA and the left IIA. This led to the patient undergoing Y-grafting and disconnection of the CIA and IIAA. Contrast-enhanced computed tomography (CT) revealed a giant IIAA, with a maximum short diameter of 66 mm, and no signs of endoleak (Fig. 1). The aneurysm presented as a cluster of three separate lobes, resembling a dumpling-like structure.

**Figure 2:**
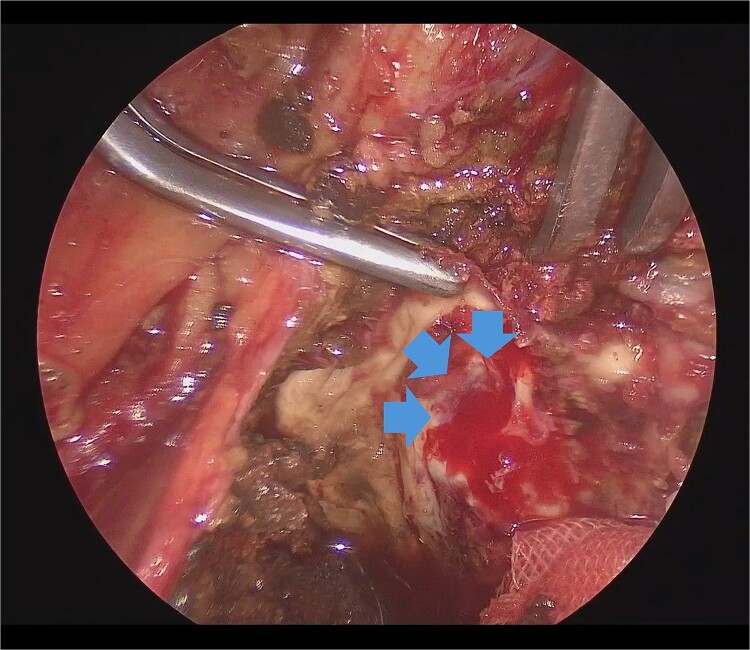
Laparoscopic view of the orifice of the feeding vessel (blue arrow).

**Figure 3:**
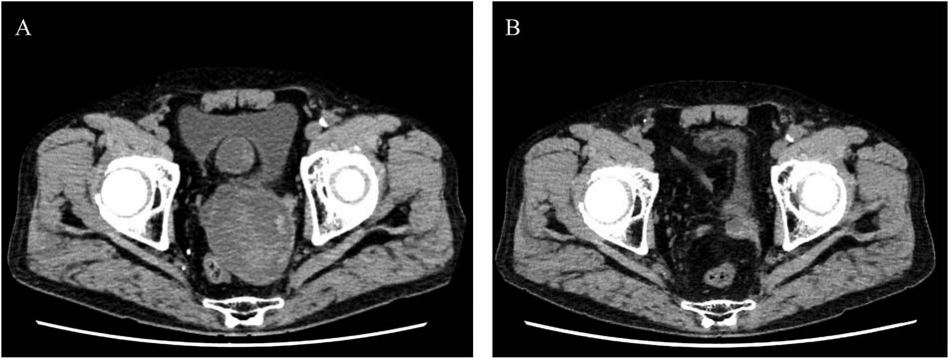
Postoperative computed tomography showing a reduction in aneurysm size. A—Preoperative image, B—10-month postoperative image.

He underwent surgery under general anesthesia. A midline abdominal incision and an intraperitoneal approach were employed. Due to the aneurysm’s size, combined with adhesions to the surrounding intestines and the thinness of the aneurysm wall, achieving complete exposure was particularly challenging. Therefore, the aneurysm was incised midway. The hematoma and coils were removed, and the feeding vessels were ligated with 3–0 monofilament. Blood oozing from the feeding vessels suggested that the aneurysm expansion was likely due to a type II endoleak that was not visible on contrast-enhanced CT. The laparoscope was used to observe the aneurysm, identify, and ligate the inflow vessels that were difficult to visualize directly (Fig. 2). The IIA was sutured with 3–0 monofilament using laparoscopic assistance, taking care to avoid including adjacent structures such as the rectum in the sutures. The operation took 255 min, and the patient was discharged on the fourteenth day postoperation. Follow-up CT at 10 months postoperation showed a reduction in aneurysm size (Fig. 3).

## Case 2

**Figure 4:**
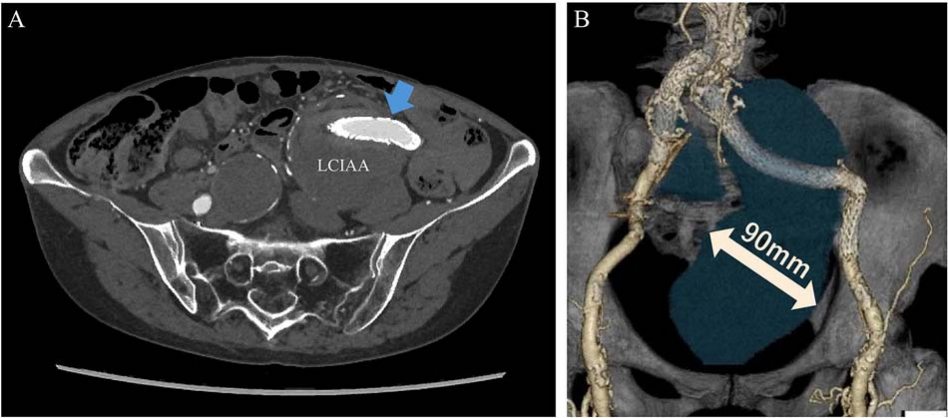
Preoperative computed tomography imaging showing a left common iliac artery aneurysm and internal iliac artery aneurysm with a maximum short diameter of 90 mm. A—Axial image, B—3D image. LCIAA, left common iliac artery aneurysm.

An 80-year-old female was admitted for the expansion of a left CIAA and IIAA. Ten years prior, she had undergone coiling of peripheral branches of the IIAA and stent graft placement in the CIAA. Contrast-enhanced CT showed the IIAA and CIAA extending into the deep pelvis. The maximum short diameter was 90 mm, and there were no signs of endoleak (Fig. 4).

**Figure 5:**
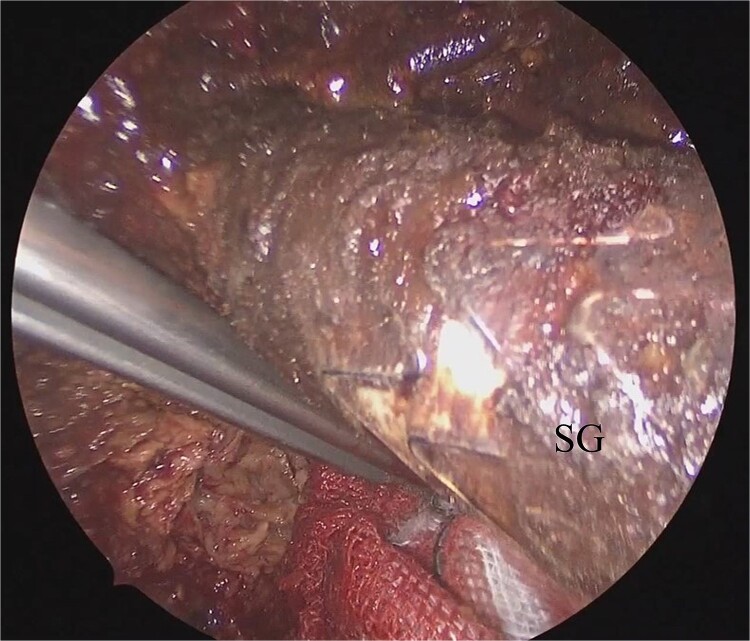
Laparoscopic view of checking for type I endoleak presence. SG, stent graft.

**Figure 6:**
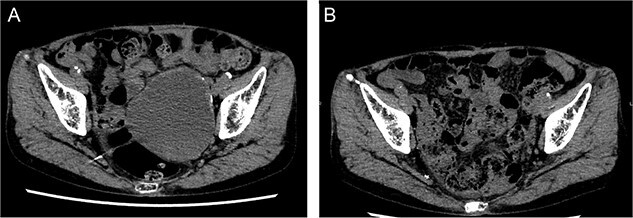
Postoperative computed tomography showing a reduction in aneurysm size. A—Preoperative image, B—7-month postoperative image.

She underwent surgery under general anesthesia. A midline abdominal incision and an intraperitoneal approach were employed. The retroperitoneal space was accessed from the lateral side of the sigmoid colon to reach the CIA. The aneurysm was incised midway. The hematoma and coils were removed, and the stent graft was observed. The laparoscope was utilized to inspect areas obscured from direct view, such as behind the stent graft, to confirm that no type I or IIIb endoleak was present (Fig. 5). A type II endoleak was observed near the proximal neck of the stent graft, which was thought to be the cause of the aneurysm expansion. The IIAA was sutured with 3–0 monofilament, and the aneurysm was closed. The left ureter was close, and care was taken during suturing to avoid including it in the stitches. The operation took 177 min, and the patient was discharged on the tenth day postoperation. Follow-up CT at 7 months postoperation showed a reduction in aneurysm size (Fig. 6).

## Discussion

Endovascular repair is commonly used for treating IAA due to its safety [2]. However, despite treatment, endoleaks were observed in 31.2% of cases and 12.5% showed aneurysm enlargement [3]. Aneurysmorrhaphy offers radical treatment, though managing bleeding from peripheral branches is challenging, especially in deep aneurysms. Ideally, feeding vessels should be ligated externally before incising the aneurysm. However, this is often impractical due to adhesions involving the intestines and ureter. If retrograde blood flow from peripheral branches is not adequately controlled, incising the aneurysm can be hazardous. Initiating treatment with endovascular procedures before advancing to aneurysmorrhaphy represents a practical strategy. If the aneurysm expands, the wall becomes thin, necessitating meticulous suturing to avoid the underlying intestines and ureter. It is important to understand the anatomical relationships preoperatively using CT imaging.

The treatment criteria for the expansion of IAA following EVAR remain poorly defined, with no substantial literature providing clear guidelines. After endovascular treatment, particularly with aneurysm expansion due to type II endoleak, the rupture risk is considered lower than the aneurysm’s diameter suggests [4]. Since IAA often shows minimal symptoms, early surgical intervention is difficult to justify [5]. However, in the two cases presented, it appears that intervention was delayed until the aneurysms had reached an enormous size, which may have contributed to the increased complexity of treatment. Waiting for the aneurysm to enlarge sufficiently to ensure adequate working space is an option, but larger aneurysms increase the risk of adhesions and aorto-enteric fistula [6]. If the aneurysm enlarges, the landing zone of a stent graft placed in the CIA may decrease, potentially leading to a type I endoleak. Once it occurs, the risk of rupture significantly increases. A comprehensive evaluation, considering the aneurysm’s diameter, shape, and stent graft position, is imperative for determining the timing of surgery.

In these cases, laparoscopy improved the procedure’s quality. The benefits of laparoscopic surgery include magnification, reduced blind spots, and a shared view for the surgeon. It was useful for observing the deep aneurysm wall and the underside of the stent graft ends. This approach likely reduces oversight in ligating feeding vessels and endoleak, making the surgeries more radical. Suturing in deep spaces under laparoscopic view avoids leaving cavities. There have been no reports yet of using laparoscopy for aneurysmorrhaphy in the iliac artery aneurysm. Using laparoscopy as an adjunct in aneurysmorrhaphy enhances surgical quality.

## Data Availability

Not applicable.
